# Update on DNA-Double Strand Break Repair Defects in Combined Primary Immunodeficiency

**DOI:** 10.1007/s11882-020-00955-z

**Published:** 2020-07-09

**Authors:** Mary A. Slatter, Andrew R. Gennery

**Affiliations:** 1 Paediatric Immunology and Haematopoietic Stem Cell Transplantation, Great North Children’s Hospital, Clinical Resource Building, Floor 4, Block 2, Newcastle upon Tyne, UK; 2 Translational and Clinical Research Institute, Newcastle University, Newcastle upon Tyne, UK

**Keywords:** Ataxia-telangiectasia, Nijmegen breakage syndrome, DNA-PK, DNA ligase 4, Cernunnos-XLF, Radiosensitivity

## Abstract

**Purpose of Review:**

The most serious DNA damage, DNA double strand breaks (DNA-dsb), leads to mutagenesis, carcinogenesis or apoptosis if left unrepaired. Non-homologous end joining (NHEJ) is the principle repair pathway employed by mammalian cells to repair DNA-dsb. Several proteins are involved in this pathway, defects in which can lead to human disease. This review updates on the most recent information available for the specific diseases associated with the pathway.

**Recent Findings:**

A new member of the NHEJ pathway, PAXX, has been identified, although no human disease has been associated with it. The clinical phenotypes of Artemis, DNA ligase 4, Cernunnos-XLF and DNA-PKcs deficiency have been extended. The role of haematopoietic stem cell transplantation, following reduced intensity conditioning chemotherapy, for many of these diseases is being advanced.

**Summary:**

In the era of newborn screening, urgent genetic diagnosis is necessary to correctly target appropriate treatment for patients with DNA-dsb repair disorders.

## Introduction

There are a number of recognized immunodeficiency syndromes due to defects in genes important for DNA-dsb repair and variable, diversity and joining (VDJ) recombination during T- and B lymphocyte formation. This review aims to provide an update on the known disorders including the molecular pathways that are involved, the clinical features and the importance of diagnosis. The immunodeficiency associated with these disorders may be amenable to treatment by haematopoietic stem cell transplantation (HSCT) although features out with the haematopoietic system will remain unchanged. Due to sensitivity to DNA damaging chemotherapeutic agents, specific approaches to transplant are required.

## Case Report

A female infant presented at the age of 12 weeks with skin rash and failure to thrive. She had no dysmorphic features and had a normal head circumference. She was lymphocytopenic and a diagnosis of severe combined immunodeficiency (SCID) was made. Her lymphocyte subsets were as follows:

CD3+ 474, CD19+ 8, CD4+ 187, CD8+ 137, NK 269 (all cells/μl) with absent naïve T-lymphocytes. She had absent IgM and IgA.

Genetic studies to define the underlying disorder were arranged, but in the interim at 8 months of age, in the absence of a matched family donor or well matched unrelated donor, she underwent a paternal haploidentical CD3 + TCR αβ/CD19+ depleted HSCT with standard conditioning according to the IEWP of EBMT guidelines of treosulfan 36 g/m^2^, fludarabine 160 mg/m^2^, thiotepa 10 mg/kg with ATG and rituximab. She developed moderate mucositis, capillary leak and chemotherapy-related skin rash and engrafted rapidly with 1st day of neutrophils above 0.5 × 10 [9]/l on day +9 and 100% donor chimerism. She developed stage III skin graft versus host disease, treated with high dose steroids and cyclosporine, and on day +39 post HSCT was found to have adenoviraemia treated with cidofovir. Simultaneously she had features of thrombotic microangiopathy (with hypertension, thrombocytopenia, low haptoglobins, renal dysfunction and elevated urine protein/creatinine ratio). This was accompanied by gastrointestinal bleeding, followed by respiratory distress with pleural and pericardial effusions. She received treatment with defibrotide and eculizumab but sadly deteriorated and died 3 months after HSCT.

During this period an unexpected diagnosis of DNA Ligase 4 deficiency was made on the basis of Sanger sequencing after an anomaly in the gene was suggested by experimental whole exome sequencing. She had compound heterozygous mutations leading to p.Arg278Pro, p.Glu582Aspfs. Subsequent radiosensitivity studies showed that her cells were exquisitely sensitive to relatively small doses of radiation suggesting her condition was at the severe end of the spectrum of disease seen with DNA Ligase 4 deficiency.

Although HSCT-related mortality for this condition remains very high regardless of the conditioning regimen used, had the diagnosis been made prior to HSCT, the conditioning chemotherapy would have been modified. This case highlights the absolute necessity for rapid genetic results to be available to inform clinicians on appropriate treatment, together with the need for newborn screening for SCID.

## Molecular Pathways

In order to generate the vast number of antigen specific receptors required to counter any invading pathogen, T- and B-lymphocytes stochastically rearrange gene segments from Variable, Diversity and Joining gene clusters, in a process known as VDJ recombination. This diversity combined with imprecise gene segment junctional alignment, and random insertion or deletion of nucleotides at the gene segment junctions, enables the creation of over 10^12^ unique T- and B lymphocyte receptors, with most diversity focused on the antigen capture region of the lymphocyte receptor.

**Fig. 1:**
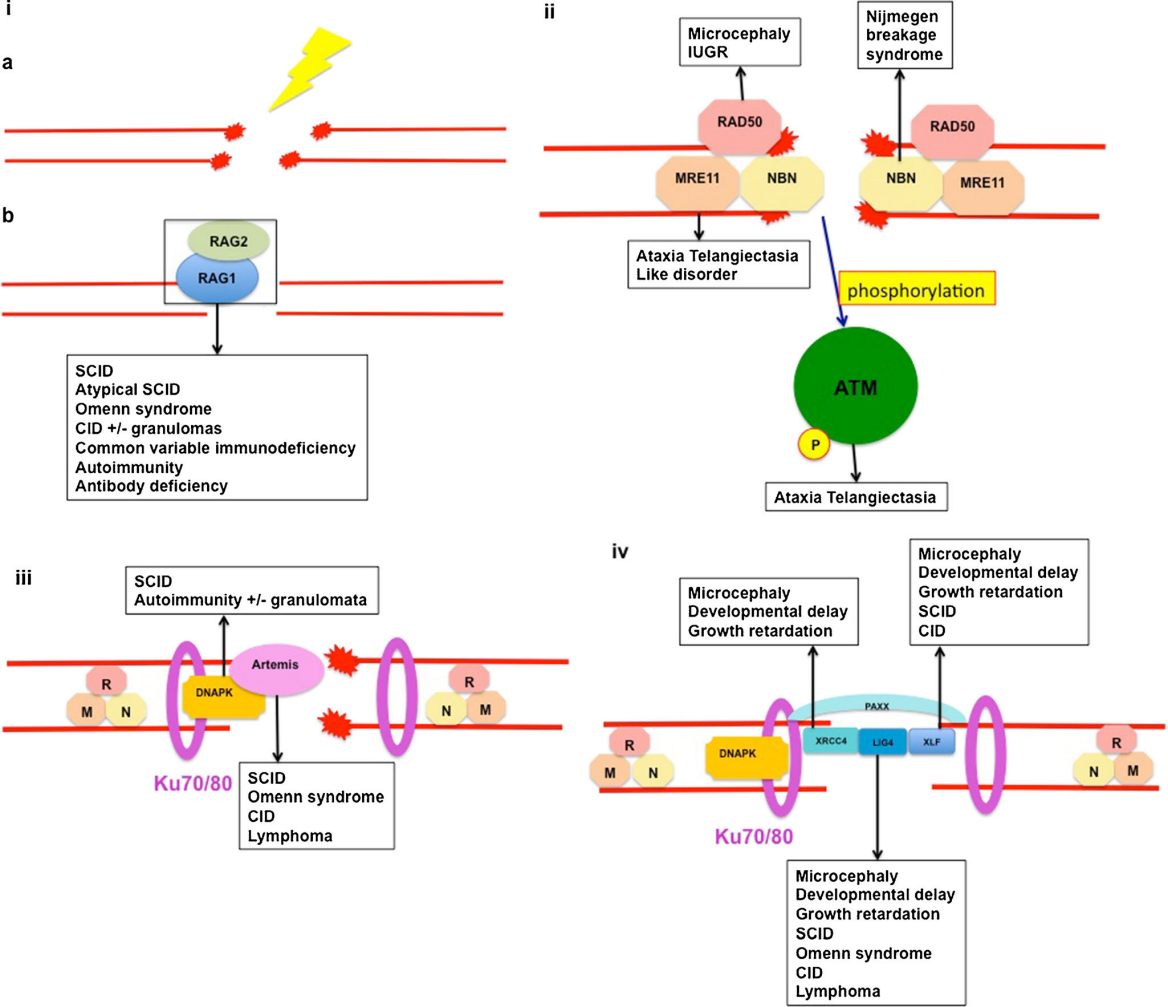
DNA double strand break repair by non-homologous end joining. DNA double strand break induced by exogenous causes such as ionizing radiation (ia) or endogenous causes such as intermediate steps in normal metabolic processes including DNA replication and meiotic recombination or physiological adaptive immune system development (ib). The MRN protein complex (MRE11, RAD50 and NBN) binds broken DNA ends and phosphorylates ataxia-telangiectasia mutated kinase (ATM), which initiates cell-cycle arrest and attraction of numerous repair proteins (ii). Ku70/Ku80 heterodimer binds the broken DNA coding ends and recruits DNA-PKcs and Artemis, which is essential to open the DNA hairpin intermediates. The covalently sealed DNA hairpin intermediate is randomly nicked by the DNA-PKcs/Artemis complex, to generate a single-stranded DNA break with 3′ or 5′ overhangs (iii). XRCC4, DNA ligase 4, Cernunnos-XLF and PAXX co-associate and are recruited to the modified DNA ends. DNA ligase 4 directly repairs the damage - the XRCC4/Cernunnos-XLF/PAXX support the enzyme (iv)

Initiation of this process is achieved by breaking the double-stranded DNA, to create DNA double strand breaks (DNA-dsb), in order to access and isolate different gene segments, prior to the assembly of the antigen receptor gene product. The lymphoid specific genes recombination activating gene (RAG)1 and 2 are responsible for initiating these DNA-dsb. Defects in *RAG1/2* lead to a number of combined immune deficiencies including T-B- NK+ SCID, combined immunodeficiencies (CID) and more mild forms of immunodeficiencies including IgA deficiency [1]. Repair of these DNA-dsb is performed by the ubiquitous DNA repair machinery found in all nucleated cells. Cells are constantly exposed to exogenous and endogenous DNA damaging agents. Unrepaired, damage to DNA can lead to replication errors, loss or rearrangement of genomic material, mutations or cancer and eventual cell death. In order to solve this, a number of DNA repair pathways have evolved. A particularly serious form of DNA damage is DNA-dsb, which can be a result of irradiation as well as physiological damage during lymphocyte receptor development (Fig. 1i). Two pathways are important to resolve the damage and maintain genome stability following DNA-dsb. In mammalian cells, information from a homologous template on sister chromatids is used to accurately repair breaks, in a process known as homologous recombination, and is generally restricted to the late S phase and G2 phase of the cell cycle. In vertebrate cells, the major DNA-repair pathway that facilitates the joining of regions of DNA that lack extensive homology is the non-homologous end-joining (NHEJ) pathway which is predominantly active during the G1 phase, but can operate at any phase of the cell cycle [2]. T- and B- lymphocytes utilize the ubiquitous NHEJ pathway to repair RAG-initiated DNA-dsb during the rearrangement of antigen receptor gene segments.

A number of proteins are involved in the NHEJ repair pathway, and are conserved through evolution, indicating the critical role they play in maintaining genomic stability. Defects in a number of these proteins have been described which cause human disease. Many of these diseases include combined immunodeficiency as part of the phenotype. However given the ubiquitous nature of the repair pathway in mammalian cells, many other non-immunological clinical features may be apparent in diseases caused by defects in these genes, and may be implicated in carcinogenesis.

### MRN Complex

The meiotic recombination 11 homologue 1 (MRE11), RAD50 and Nijmegen breakage syndrome protein 1 (NBS1) proteins play a pivotal role in sensing DNA-dsb and coordinating the response to initiate cell cycle checkpoint arrest and commence DNA repair or initiate apoptosis. This compound (the MRN complex), which exhibits dual single strand DNA endonuclease and double strand DNA exonuclease activity, comes together as a heterodimer complex to execute three indispensable functions in DNA-dsb repair:

- binding and processing of damaged DNA
- securing DNA to bridge over short and long distance damage regions
- activation of DNA damage response and checkpoint signalling pathways [3] (Figure 1ii).

Human disease has been described due to mutations in *MRE11* (Ataxia-Telangiectasia-like disorder, OMIM #604391) [4–6], *RAD50* (Nijmegen Breakage Syndrome-like disorder) [7•,^8^] and *NBN*, and although the somatic phenotype shows some common features, significant immunodeficiencies are confined to patients with *NBN* mutations giving rise to Nijmegen Breakage syndrome (NBS) (OMIM #251260).

### Ataxia Telangiectasia Mutated

The activated MRN complex initiates the cell cycle checkpoint response by promoting the localized activation of ataxia-telangiectasia mutated (ATM) protein, which is a central component of the signal transduction pathway through a variety of cellular signalling pathways in response to DNA damage, including cell cycle control, apoptosis, senescence, transcription, chromatin structure alteration and DNA repair. Activated ATM phosphorylates the MRN complex, resulting in cascade of phosphorylation of hundreds of ATM substrates [9] (Figure 1ii). Whilst the majority (~80%) of irradiation-induced DNA-dsbs are repaired by the NHEJ pathway independently of ATM, a minority are repaired by a pathway requiring ATM and Artemis [10]. Defects in *ATM* give rise to ataxia-telangiectasia (AT) (OMIM #208900).

### Non-Homologous End Joining

The NHEJ repair pathway for DNA-dsbs has three aims:

- synapsis of two broken DNA ends
- end processing to make them possible to ligate
- ligation of these ends together.

A series of eight proteins have been identified as the critical NHEJ components, which are involved in the ligation of DNA-dsb [11]. The DNA-binding subunits Ku70 and Ku80 together form a ring shaped heterodimer that acts as an anchor protein binding the DNA ends and protecting them from exonucleolytic activity. A single Ku heterodimer binds to each DNA end, and interacts with DNA protein kinase catalytic subunit (DNA-PKcs) to form a holoenzyme, DNA-PK. DNA-PK acts as a bridge between two Ku heterodimer-bound DNA ends, acting to stabilize the local DNA structure to enable end-processing and DNA ligation. The Ku enzymes have not yet been implicated in human disease. Mutations in *PRKDC,* which encodes the DNA protein kinase catalytic subunit lead to CID (OMIM #615966).

Artemis, encoded by *DCLRE1C*, is phosphorylated by activated DNA-PKcs, which initiates the endonuclease activities of Artemis allowing resolution of complex DNA ends including the heterologous loop and stem-loop DNA structures that contain single-stranded DNA adjacent to double-stranded DNA (Figure 1iii). Defects in *DCLRE1C* lead to a number of immunodeficiencies including SCID and CID (OMIM #602450). DNA ligase 4, XRCC4 and Cernunnos-XRCC4-like factor (XLF) act as link proteins, bridging the DNA ends – DNA ligase 4 is required for the ligation reaction that rejoins the DNA-dsbs. DNA ligase 4 (OMIM #606593) and cernunnos-XLF (OMIM #611291) have been implicated in human immunodeficiency. Patients with XRCC4 deficiency are described (OMIM #616541), but although short stature and microcephaly are features, immunodeficiency has not been described. Most of the NHEJ process functions separately from ATM signalling, although a fraction contingent upon Artemis requires ATM activity, demonstrating some relationship between the signalling and repair machinery.

The most recent factor involved in NHEJ to be described is Paralog of XRCC4 and XLF (PAXX), which has a similar structure to XRCC4, and interacts and binds with Ku, stabilizing the NHEJ protein assembly [12••, 13••, 14••] (Figure 1iv). To date, no human disease has been described involving PAXX, and it is not clear whether defects have any impact on adaptive immunity.

## Combined Immunodeficiencies Associated with Defects in DNA Double Strand Break Repair

As indicated above, defects in a number of proteins critical for DNA-dsb sensing and repair confer human disease (Table 1). Many of these are associated with immunodeficiency, and all display sensitivity to ionizing radiation.

**Table 1:** Disorders of non homologous end joining DNA double strand break repair

Disorder	Pathway	Clinical features
	Gene mutations	
	Inheritance	
Artemis	NHEJ	1.T-B-NK+ SCID - Early infancy viral infection, PJP, Diarrhoea and FTT
	Null mutations in *DCLRE1C*	2. Omenn syndrome
	AR	3. Atypical late onset SCID - Recurrent infection, AI, EBV-lymphoma
Ligase 4 deficiency	NHEJ	SCID or atypical SCID, Omenn syndrome, CID, asymptomatic lymphocytopenia, malignancy, marrow hypoplasia, malignancy
	Hypomorphic mutations in *LIG4*	
	AR	
		May have microcephaly and growth failure.
Cernunnos-XLF	NHEJ	Microcephaly, learning difficulty, growth failure, SCID or CID
	Hypomorphic mutations in *NHEJ1*	
	AR	
DNA-PKcs	NHEJ	SCID
	Defects in *PRKDC*	AI, granulomata, microcephaly
	AR	
XRCC4 Deficiency	NHEJ	Microcephaly, growth retardation and developmental delay
	Mutations in *XRCC4*	
	AR	No significant immunodeficiency
Ataxia Telangiectasia	MRN complex	Progressive cerebellar ataxia, oculocutaneous telangiectasia, infertility, growth retardation, lymphoid tumours, recurrent infection, chronic lung disease
	Ataxia-telangiectasia mutated (ATM) protein defects	
	AR	
Nijmegen Breakage Syndrome	MRN complex	Dysmorphic facies, IUGR, growth retardation
	*NBS1* mutations	
	AR	Skeletal and renal abnormalities, mental retardation
		Recurrent sino-pulmonary infection, B-lymphoma, AI
Ataxia telangiectasia-like disorder	MRN complex	Similar to AT, but no telangiectasia. Ataxia later and milder
	Mutations in *MRE11*	
	AR	
RAD 50	MRN complex	IUGR, microcephaly, and poor postnatal growth
	Mutations in *RAD50*	No significant immunodeficiency
	AR	

Abbreviations: NHEJ, non homologous end joining; AR, Autosomal recessive; SCID, Severe Combined Immunodeficiency; PJP, *Pneumocystis jirovecii* pneumonia; FTT, failure to thrive; AI, Autoimmunity; Lig 4, Ligase 4; CID, Combined Immunedeficiency; XLF, XRCC4-like factor; XRCC4, X-ray cross-complementation group 4; DNA-PKcs, DNA-dependent protein kinase subunit; PRKDC, Protein kinase catalytic subunit; MRN, Complex of 3 proteins – Mre11, Rad50, NBS1; NBS1, Nijmegen Breakage Syndrome protein 1; IUGR, Intrauterine growth retardation; MRE 11, Meiotic recombination 11

### Artemis Deficiency

Artemis is critical for VDJ recombination, demonstrated by null mutations in *DCLRE1C* leading to a T-B-NK+ SCID phenotype with absent immunoglobulins [15], which was initially described in Athabascan-speaking native Americans [16]. Patients present as with any other form of SCID, classically in early infancy with viral or pneumocystis pneumonitis, persistent viral diarrhoea and growth failure [17]. There is a systemic increased cellular sensitivity to ionizing radiation. Although SCID is the most common presentation, other clinical phenotypes are described including atypical SCID with hyper IgM [18•], Omenn syndrome [19], progressive CID presenting from later infancy and typified by recurrent infection of the gastrointestinal or sino-respiratory tracts, T- and B-lymphocytopaenia, hypogammaglobulinaemia and autoimmune cytopaenias, some with EBV-associated B lymphomas [20, 21], and antibody deficiency with a common variable immunodeficiency phenoytype [22]. Autoimmunity is commonly described in the non-SCID presentations [23]. The severity of the clinical phenotype correlates with the levels of recombination and DNA repair activity conferred by the protein, determined by the type and genetic locus of the mutation [24]. Microcephaly is not a feature of *DCLRE1C* mutations.

The outcome of HSCT in patients with SCID is significantly better in those transplanted without pre-existing infection [25, 26]. This observation has directed the institution of newborn screening programs for SCID in many states in the USA [27], by identification of DNA remnants present in lymphocytes, and left over following successful VDJ recombination, revealing successful T lymphocyte receptor re-arrangement (T lymphocyte receptor excision circles – TRECs). T lymphocyte neo-genesis, successful thymopoiesis and durable T lymphocyte engraftment, as well as probability of B lymphocyte function requires haematopoietic stem cell engraftment, most likely achieved by use of preparative chemotherapy [28, 29]. The use of alkylating-containing conditioning regimens confers longterm immune-reconstitution in patients with *DCLRE1C* mutations [30], but leads to significant long-term sequalae [31], and possibly an increased risk of early conditioning-related mortality [32•]. These factors have driven the quest for safer alternatives to achieve functioning immunity. Two promising developments which have entered clinical trials are lentiviral-vector gene-addition therapy [33••, 34••] and the use of chemotherapy free antibody-based conditioning [35]. Longterm results are awaited, but preliminary results are encouraging.

### Ataxia Telangiectasia

**Fig. 2:**
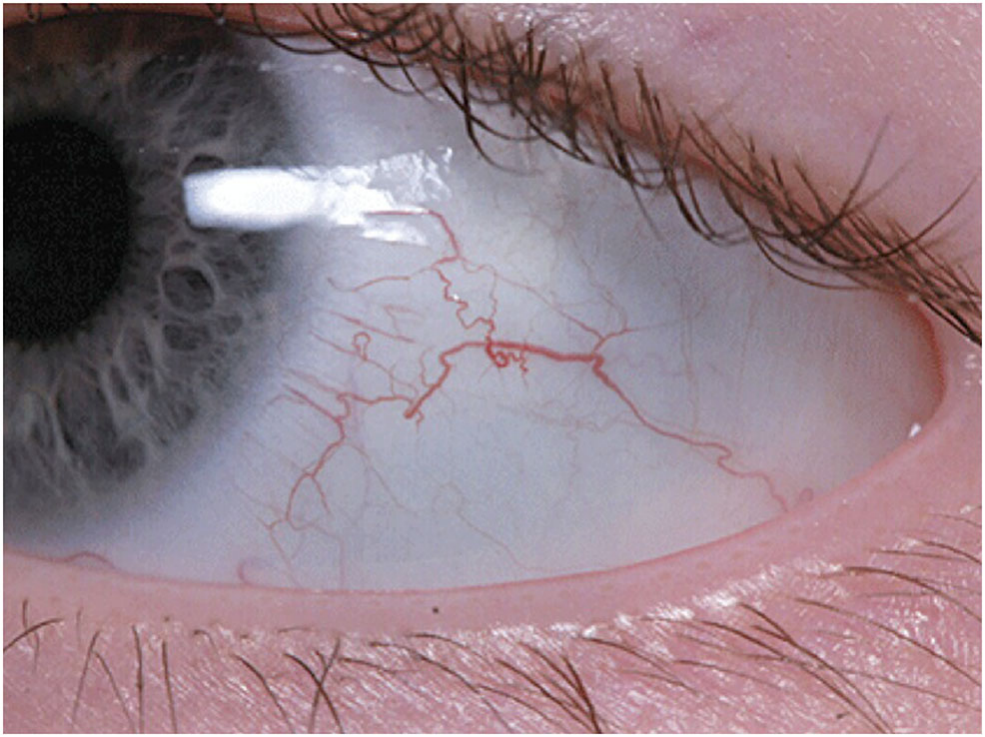
Early bulbar telangiectasia on a 2.5 year old patient with ataxia-telangiectasia

AT is a rare systemic disorder, inherited in an autosomal recessive fashion, and caused by mutations in *ATM* [36]. Systemic clinical features include progressive cerebellar ataxia, oculocutaneous telangiectasia (Fig. 2), gonadal sterility, and growth retardation. Patients experience a high frequency of lymphoid tumours. Immunodeficiency may lead to frequent sinopulmonary infections that in conjunction with recurrent aspiration, lead to chronic lung disease [37]. Interstitial lung disease, including lymphoid interstitial pneumonitis is also reported. Patients with AT have a variable incidence of infections;- some experience no more than unaffected siblings, whereas others manifest progressive respiratory infection because of humoral and cellular immunodeficiency. Immune responses, especially to bacterial polysaccharide antigens, are generally reduced [38]. Mutations in *ATM* do not lead to a significant block in lymphocyte development. Constancy of VDJ recombination rather than completion of VDJ recombination may be affected in the absence of ATM, which may be important to monitor recombination intermediate products. IgA, IgE and IgG subtypes are reduced or absent in AT patients and, in some, IgM may be raised, which may represent a more severe immunological defect with worse prognosis [39–41]. Heterozygote carriers do not display the classical manifestations of disease, but may have a higher incidence of solid tumours, particularly breast cancer in female carriers [42]. Ataxia is normally the earliest clinical manifestation of AT [43]. Most infants appear to be normal during the first year of age - walking develops normally, although some demonstrate ataxia in infancy with a delay in walking. Abnormal eye movements generally develop from the second year of life onwards [44, 45] and oculocutaneous telangiectasias appear on the bulbar conjunctivae and exposed areas of the skin after the neurological deficit has manifest. Café-au-lait patches are also common.

Patients with AT have a significantly increased risk of developing malignancy - from 10 years of age, the risk of developing a tumour is around 1% annually. Around 25% will develop malignancy, of which lymphoma and leukaemia predominate [46•]. Malignancy may be difficult to treat – the tumours are often aggressive, and because of the systemic nature of the disease, significant morbidity and mortality to chemotherapy and radiotherapy may be experienced. Currently there are no curative therapies for patients with AT, median age of death is 25 years [47], and older patients have significant multi-system co-morbidities [48••].

There is a tentative suggestion that chronic inflammatory processes, driven in part by persistent genotoxic stress, participate in the pathogenesis in AT [49, 50]. Furthermore, in addition to the intrinsic genomic instability, impaired immune surveillance may contribute to tumorogenesis and development. Murine Atm-deficient T-lymphocytes demonstrate impaired proliferative capacity because of replication stress [51]. These observations raise the possibility that modification of the immune system may alleviate some of the disease sequelae. Haematopoietic stem cell transplantation has been considered for AT patients. Use of fully myelo-ablative conditioning is associated with significant morbidity and mortality [52••]. The adoption of a reduced intensity conditioning regimen gives better survival and enables donor engraftment and immune reconstitution [53–55•]. The long-term outcome with respect to systemic damage to tissues has yet to be determined, and there may be an increase in secondary malignancies, as there is for Fanconi anaemia patients who have been transplanted [56]. Furthermore, the efficacy of HSCT in terms of ameliorating damage secondary to inflammation, and malignancy due to enhanced tumour surveillance are unproven to date. Adoption of HSCT for patients with HSCT would best be undertaken in the context of clinical trials, with careful long-term follow up.

Newborn screening for SCID detects TRECs, the by-product of successful T lymphocyte development. Significant T-lymphocytopenia will therefore be detected by TREC screening, and non-SCID conditions may be picked up incidentally, including patients with AT [57•, 58•]. This presents an ethical dilemma as:

- Potentially the infant may undergo HSCT, which is not appropriate
- If standard conditioning protocols are used, there is a significant risk of morbidity and mortality
- Families may not wish to be informed that their newborn infant has a progressive, fatal neurodegenerative disease
- A positive diagnosis of AT in the infant is likely to implicate the mother as a carrier, who will need to be counselled about increased risks of developing a tumour, and in particular, breast cancer.

The first two points can be resolved by ensuring that a genetic diagnosis is available prior to embarking upon a stem cell procedure. The final points require sensitive counselling before further testing is performed. Many parents would prefer to have information regarding an early diagnosis of AT, although a significant minority would prefer not to know [59••,^60^].

### Nijmegen Breakage Syndrome

**Fig. 3:**
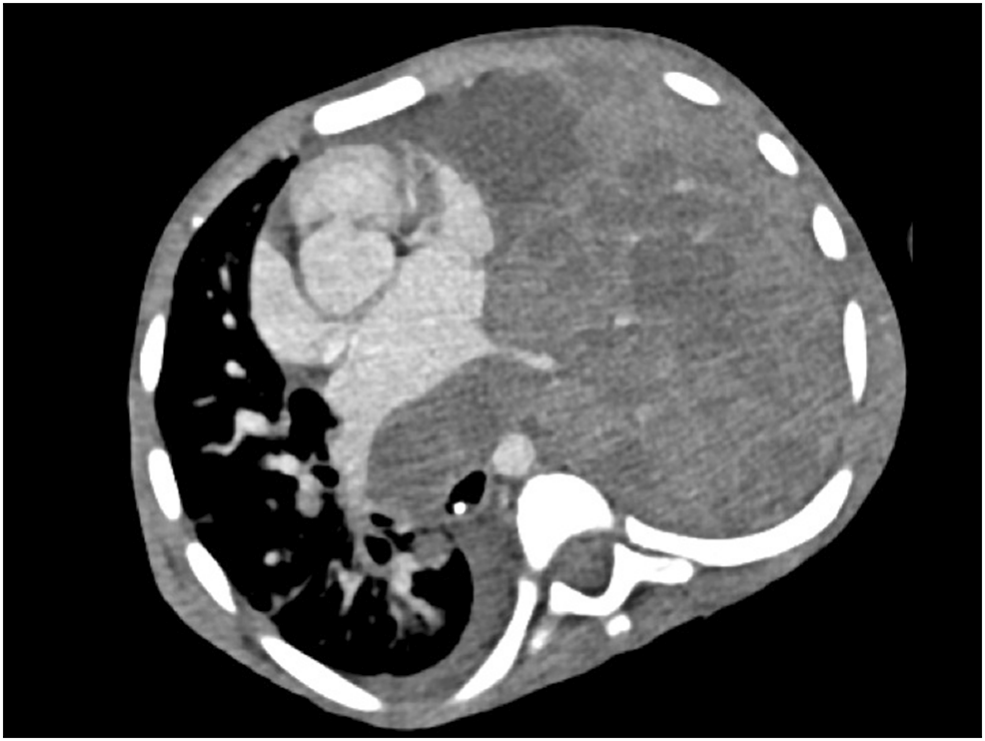
Rapidly progressive left sided thoracic non-Hodgkin lymphoma in an 8 year old patient with Nijmegen Breakage syndrome

Nijmegen breakage syndrome (NBS) is typified by a characteristic dysmorphic facial appearance, which becomes exaggerated with advancing age. Intrauterine growth retardation is usually present and patients exhibit profound microcephaly at birth. They display a receding forehead, receding mandible and prominent midface [61]. Other features include short stature, congenital skeletal (clinodactyly, syndactyly) and renal abnormalities, and mild non-progressive mental retardation. Premature ovarian insufficiency is reported in females. Although reported worldwide, there is a particularly high prevalence among Central and Eastern European Slavic populations due to a founder mutation effect (a homozygous deletion of five nucleotides (657_661del5)) [62]. Occasional patients may have normal head size, although display other features typical of NBS [63]. Sino-pulmonary infection is common and patients are susceptible to malignancies, particularly B lymphocyte-lineage lymphomas (Fig. 3), and autoimmune manifestations, including pulmonary granulomata and interstitial lymphocytic lung disease [64, 65]. Cellular and humoral immune deficiency are widely reported, but with a spectrum of clinical expression ranging from clinically-silent laboratory abnormalities (reduced CD4+, CD8+ T-lymphocytes, thymic emigrants, low percentage of naïve T-lymphocytes, increased memory T-lymphocytes, reduced TCRαβ/ TCRγδ ratio in peripheral blood) to clinically relevant immunodeficiency, particularly hypogammaglobulinaemia, which presents with recurrent, chronic respiratory tract infections. This may cause development of bronchiectasis, and many patients require immunoglobulin substitution therapy. Opportunistic infections are rare and there is generally no correlation between the degree of cellular deficiency and infection severity of infections [66]. Some patients with Nijmegen breakage syndrome may be identified on newborn screening for SCID with very low levels of TRECs [67•].

Malignancy remains the most significant risk for patients with NBS, with most tumours arising from the lympho-reticular system. By 20 years of age, over 40% of patients have acquired a malignancy, and the median age at which malignancy develops is 10 years [68]. Because the repair defect is systemic, patients often fail to tolerate treatment, and have a higher frequency of chemotherapy-related toxicity, and severe or fatal infectious complications during chemotherapy than observed in otherwise healthy children receiving chemotherapy. The adverse response to treatment results in a high rate of treatment failure and increased risk of developing secondary malignancies.

Given these issues, the arguments for considering HSCT to treat patients with NBS are perhaps more compelling than for patients with AT – the neurological problems associated with NBS are not progressive, and many early deaths may be prevented by altering immune surveillance. Regimens using reduced intensity conditioning or Fanconi-anaemia type protocols have resulted in successful outcomes [52••, 62, 64, 69]. The role of pre-emptive HSCT has yet to be formalized, but could be contemplated before malignancy develops, particularly in patients with clinical immunodeficiency, recurrent or chronic infection despite immunoglobulin therapy, latent viral infection (especially EBV) or severe autoimmunity. However, careful long-term follow up is required to determine whether the incidence of secondary malignancy might be lower than that recorded in non-transplanted individuals. One report documents a loss of donor myeloid chimerism in patients receiving reduced intensity conditioning, but improved chimerism when treosulfan was added to the regimen [70]. Follow up was short, however, at only 3 years and longer follow up is required to determine whether the risk of secondary malignancy is increased with the higher intensity regimen.

### Ligase 4 Deficiency and Cernunnos/XLF Deficiency

A few patients have been described with hypomorphic mutations in *LIG4*. Most have microcephaly, although individuals with a normal head circumference are described [71]. The spectrum of presentation in individuals with LIG4 deficiency is wide with cases of SCID or atypical SCID [72, 73], Omenn syndrome [74], CID [75, 76], asymptomatic CD4+ T-lymphocytopenia [77], predisposition to malignancy [71], marrow hypoplasia and even asymptomatic individuals [78••], some of whom display microcephaly and growth failure [79]. As well as immunodeficiency, patients are at significant risk of developing malignancies, predominantly lymphomas or leukaemias, often, but not always associated with EBV [71, 75, 80]. Whilst microcephaly and growth failure are characteristic, they are not universally present, and the clinico-immunological phenotype is indistinguishable from that of patients with NBS. T-lymphocytopenia is often present, and peripheral B-lymphocytes may be almost absent. A high IgM and low IgA and IgG may be seen, due to the role of LIG4 in class switch recombination. Treatment is expectant, with antimicrobial prophylaxis and immunoglobulin replacement as required. Treatment of malignancy is difficult, as tumours are often aggressive and the systemic distribution of the defect means that patients are poorly tolerant of chemotherapy and radiotherapy [71]. The role of HSCT is unclear. A number of patients have successfully been treated, particularly when reduced intensity conditioning regimens are employed [52••], but the optimal patient selection and timing have yet to be determined. Whilst HSCT will correct the immunodeficiency and associated marrow hypoplasia, it is unclear whether successful treatment will prevent development of malignancy or whether late-onset conditioning-related secondary malignancies will develop.

Cernunnos-XLF interacts closely with DNA ligase 4. To date, only a few patients with hypomorphic mutations in *NHEJ1* have been described. The phenotypic features are similar to those of patients with NBS and LIG4 deficiency, namely profound microcephaly with variable degrees of learning difficulty, growth failure, SCID or CID [81–83]. Lymphoma has also been described in one patient [84•]. The immunological phenotype is similar to that in patients with LIG4, namely T-lymphocytopenia with profound peripheral B-lymphocytopenia, high IgM and low IgA and IgG. A few patients have successfully been transplanted using reduced intensity conditioning regimens [52••].

### DNA-PKcs

DNA-PKcs acts early in the repair of DNA-dsb, and interacts with artemis. Only six patients with defects in *PRKDC* have been described to date, too few to confidently ascribe a typical phenotype. The first patient had a SCID phenotype, not associated with microcephaly [85]. Microcephaly has been described in one patient with SCID [86]:- other presentations have described autoimmunity with granulomata [87•, 88•]. Stem cell transplantation has been successfully attempted, but there are too few data to make any clear recommendations. It could be predicted that patients will react more like those with artemis deficiency than deficiencies of other core NHEJ proteins.

## Diagnosis

The key feature of the diseases described above is an exquisite sensitivity to ionizing radiation. However, the diagnosis of radiosensitivity is difficult, and available in only a few laboratories – furthermore, results usually take at least 4–6 weeks, as fibroblasts have to be harvested, cultured, and the experiments set up. A high index of clinical suspicion is required when contemplating the diagnosis. Some clinical and routine laboratory diagnostic signs may suggest the diagnosis. Key clinical features include telangiectasia, especially on the bulbar conjunctiva and when associated with ataxic signs, and microcephaly, particularly when associated with marrow hypoplasia, immunodeficiency, autoimmunity or lymphoid malignancy. However, even in phenotypes where microcephaly is classic, some patients may not display this sign

Alphafeto protein is raised in patients with AT.

An immunological profile that should raise a suspicion is one with a T- and B-lymphocytopaenia with preserved NK cell count, associated with a raised IgM and low or absent IgA and IgG.

During normal lymphocyte development, lymphocyte receptor gene rearrangements advance through a unique somatic recombination development. The TCRα locus uses a multi-step recombination process, using proximal TRAV elements progressively to distal TRAV elements. The persistent reduced recombinase activity over successive waves of TCRα rearrangement in patients with VDJ recombination defects is displayed as a bias in TCRα use of more proximal TRAV/TRAJ associations. These particular patterns can be detected using flow cytometric methods, which may indicate a deficit in VDJ recombination, including NHEJ defects [89•]. Cytogenetic analysis can be helpful to elucidate the diagnosis: an increased number of chromosome 7:14 translocations are seen in ataxia telangiectasia, NBS, artemis deficiency, and may be seen in the other defects described, although absence does not exclude the diagnosis.

Sensitivity to ionizing radiation can be established with a clonogenic survival assay. Fibroblasts irradiated with increasing ionizing radiation doses are assessed after 3 weeks to assess percentage survival of cells [21]. Alternatively, cells can be subject to increasing doses of radiation and subsequently stained for H2AXγ foci which cluster at the site of DSBs, but disappear over time as the DNA-dsb are repaired. Foci persistence, visualized microscopically [10] or by flow cytometry [90, 91] indicates impaired repair mechanisms. Ultimately, a confident diagnosis requires genetic analysis to identify the mutations in the appropriate gene.

## Other Diseases Caused by Molecular Defects in the NHEJ Pathway

The diseases described above are associated with T lymphocyte immune impairment, which has a significant clinical impact. Other diseases are described due to mutations in NHEJ genes where immunodeficiency is a minor issue or immunity is not impaired.

### Ataxia-Telangiectasia like Disorder

Ataxia telangiectasia-like disorder is caused by mutations in *MRE11*, part of the MRN complex which associates with NBS1, and is extremely rare, with few patients reported worldwide [92, 93•]. Clinical features mimic those of patients with AT although telangiectasia are absent and progressive cerebellar ataxia occurs later and progresses more slowly. Immunoglobulin levels are normal, but deficiency in pneumococcal polysaccharide antigen antibodies has been reported, particularly to pneumococcal polysaccharide antigen [94]. Some patients are reported to exhibit microcephaly.

### RAD50 Deficiency

Only two patients have been described with NBS-like features, in whom compound heterozygous or homozygous mutations in RAD50, the third component of the MRN complex, were found [7•,^8^]. The clinical features more resemble NBS than ATLD, with intrauterine growth retardation, microcephaly, and poor postnatal growth. There was no history of excessive infections and immunoglobulin levels were normal.

### XRCC4 Deficiency

Numerous individuals have been described with mutations in *XRCC4* [95–99]. Whilst the clinical phenotype is quite severe, with microcephaly, growth retardation and developmental delay, and there is severe radiosensitivity with a DNS-dsb defect, VDJ recombination appears preserved, with no significant immunodeficiency.

### Ku70, Ku80, PAXX

Whilst these proteins are represented in NHEJ and presumably VDJ recombination, to date, no human disease has been described attributable to defects in these genes.

## Discussion

A number of immunodeficiency syndromes are recognized due to defects in genes important for DNA-dsb repair, and necessary for VDJ recombination during T- and B lymphocyte formation. Treatment of these patients requires a specific approach, as the DNA repair defect is ubiquitous to all cells, not just lymphocytes. Therefore, use DNA-damaging agents during HSCT or treatment of malignancies may lead to specific severe morbidities and mortality. The introduction of newborn screening for SCID will detect a number of these diseases, meaning that an accurate and speedy genetic diagnosis is imperative in order to define the most appropriate treatment regimen. Furthermore, the high incidence of lymphoid malignancies in these diseases should alert clinicians treating oncology patients to the possibility of an underlying systemic DNA repair disorder indicative of primary immunodeficiency [100], prior to embarking on treatment, to minimize treatment-related toxicities.

“Online Mendelian Inheritance in Man (OMIM): https://www.omim.org“.

## References

[1] Gennery A. Recent advances in understanding RAG deficiencies. *F1000Res*. 2019;8. pii: F1000 Faculty Rev-148. 10.12688/f1000research.17056.1 PMC6364374 30800289

[2] O’Driscoll M, Jeggo PA (2006). The role of double-strand break repair – insights from human genetics. Nat Rev Genet.

[3] Bian L, Meng Y, Zhang M, Li D (2019). MRE11-RAD50-NBS1 complex alterations and DNA damage response: implications for cancer treatment. Mol Cancer.

[4] Fernet M, Gribaa M, Salih MAM, Seidahmed MZ, Hall J, Koenig M (2005). Identification and functional consequences of a novel MRE11 mutation affecting 10 Saudi Arabian patients with the ataxia telangiectasia-like disorder. Hum Molec Genet.

[5] Delia D, Piane M, Buscemi G, Savio C, Palmeri S, Lulli P, Carlessi L, Fontanella E, Chessa L (2004). MRE11 mutations and impaired ATM-dependent responses in an Italian family with ataxia-telangiectasia -like disorder. Hum Molec Genet.

[6] Stewart GS, Maser RS, Stankovic T, Bressan DA, Kaplan MI, Jaspers NGJ, Raams A, Byrd PJ, Petrini JHJ, Taylor AMR (1999). The DNA double-strand break repair gene hMRE11 is mutated in individuals with an ataxia-telangiectasia-like disorder. Cell.

[7] • Ragamin A, Yigit G, Bousset K, Beleggia F, Verheijen FW, de Wit MY, et al. Human RAD50 deficiency: Confirmation of a distinctive phenotype. *Am J Med Genet A*. 2020 (in press). **An important description of the second reported patient with RAD50 deficiency.** 10.1002/ajmg.a.61570 PMC7318339 32212377

[8] Waltes R, Kalb R, Gatei M, Kijas AW, Stumm M, Sobeck A, Wieland B, Varon R, Lerenthal Y, Lavin MF, Schindler D, Dörk T (2009). Human RAD50 deficiency in a Nijmegen breakage syndrome-like disorder. Am J Hum Genet.

[9] Blackford AN, Jackson SP (2017). ATM, ATR, and DNA-PK: the trinity at the heart of the DNA damage response. Mol Cell.

[10] Riballo E, Kuhne M, Rief N, Doherty A, Smith GCM, Recio MJ (2004). A pathway of double-strand break rejoining dependent upon ATM, Artemis, and proteins locating to gamma-H2AX foci. Mol Cell.

[11] Wu Q (2019). Structural mechanism of DNA-end synapsis in the non-homologous end joining pathway for repairing double-strand breaks: bridge over troubled ends. Biochem Soc Trans.

[12] Ochi T, Blackford AN, Coates J, Jhujh S, Mehmood S, Tamura N (2015). PAXX, a paralog of XRCC4 and XLF, interacts with Ku to promote DNA double-strand break repair. Science.

[13] Xing M, Yang M, Huo W, Feng F, Wei L, Jiang W (2015). Interactome analysis identifies a new paralogue of XRCC4 in non-homologous end joining DNA repair pathway. Nat Commun.

[14] Craxton A, Somers J, Munnur D, Jukes-Jones R, Cain K, Malewicz M (2015). XLS (c9orf142) is a new component of mammalian DNA double-stranded break repair. Cell Death Differ.

[15] Moshous D, Callebaut I, de Chasseval R, Corneo B, Cavazzana-Calvo M, Le Deist F (2001). Artemis, a novel DNA double-strand break repair/V(D)J recombination protein, is mutated in human severe combined immune deficiency. Cell.

[16] Jones JF, Ritenbaugh CK, Spence MA, Hayward A (1991). Severe combined immunodeficiency among the Navajo. I. Characterization of phenotypes, epidemiology, and population genetics. Hum Biol.

[17] van der Burg M, Gennery AR (2011). Educational paper. The expanding clinical and immunological spectrum of severe combined immunodeficiency. Eur J Pediatr.

[18] Bajin IY, Ayvaz DC, Ünal S, Özgür TT, Çetin M, Gümrük F (2013). Atypical Combined Immunodeficiency Due to Artemis Defect: A Case Presenting as Hyperimmunoglobulin M Syndrome and With LGLL. Mol Immunol.

[19] Ege M, Ma Y, Manfras B, Kalwak K, Lu H, Lieber MR, Schwarz K, Pannicke U (2005). Omenn syndrome due to ARTEMIS mutations. Blood.

[20] Moshous D, Pannetier C, de Chasseval R, le Deist F, Cavazzana-Calvo M, Romana S (2003). Partial T and B lymphocyte immunodeficiency and predisposition to lymphoma in patients with hypomorphic mutations in Artemis. J Clin Invest.

[21] Evans PM, Woodbine L, Riballo E, Gennery AR, Hubank M, Jeggo PA (2006). Radiation-induced delayed cell death in a hypomorphic Artemis cell line. Hum Mol Genet.

[22] Volk T, Pannicke U, Reisli I, Bulashevska A, Ritter J, Björkman A, Schäffer AA, Fliegauf M, Sayar EH, Salzer U, Fisch P, Pfeifer D, di Virgilio M, Cao H, Yang F, Zimmermann K, Keles S, Caliskaner Z, Güner S¸, Schindler D, Hammarström L, Rizzi M, Hummel M, Pan-Hammarström Q, Schwarz K, Grimbacher B (2015). DCLRE1C (ARTEMIS) mutations causing phenotypes ranging from atypical severe combined immunodeficiency to mere antibody deficiency. Hum Mol Genet.

[23] Lee PP, Woodbine L, Gilmour KC, Bibi S, Cale CM, Amrolia PJ, Veys PA, Davies EG, Jeggo PA, Jones A (2013). The many faces of Artemis-deficient combined immunodeficiency - two patients with DCLRE1C mutations and a systematic literature review of genotype-phenotype correlation. Clin Immunol.

[24] Felgentreff K, Lee YN, Frugoni F, Du L, van der Burg M, Giliani S (2015). Functional analysis of naturally occurring DCLRE1C mutations and correlation with the clinical phenotype of ARTEMIS deficiency. J Allergy Clin Immunol.

[25] Brown L, Xu-Bayford J, Allwood Z, Slatter M, Cant A, Davies EG, Veys P, Gennery AR, Gaspar HB (2011). Neonatal diagnosis of severe combined immunodeficiency leads to significantly improved survival outcome: the case for newborn screening. Blood.

[26] Pai S-Y, Logan BR, Griffith LM, Buckley RH, Parrott RE, Dvorak CC, Kapoor N, Hanson IC, Filipovich AH, Jyonouchi S, Sullivan KE, Small TN, Burroughs L, Skoda-Smith S, Haight AE, Grizzle A, Pulsipher MA, Chan KW, Fuleihan RL, Haddad E, Loechelt B, Aquino VM, Gillio A, Davis J, Knutsen A, Smith AR, Moore TB, Schroeder ML, Goldman FD, Connelly JA, Porteus MH, Xiang Q, Shearer WT, Fleisher TA, Kohn DB, Puck JM, Notarangelo LD, Cowan MJ, O'Reilly RJ (2014). Transplantation outcomes for severe combined immunodeficiency, 2000–2009. N Engl J Med.

[27] Kwan A, Abraham RS, Currier R, Brower A, Andruszewski K, Abbott JK, et al. Newborn screening for severe combined immunodeficiency in 11 screening programs in the United States. JAMA. 2014;312:729–38. 10.1001/jama.2014.9132 PMC4492158 25138334

[28] Hassan A, Lee P, Maggina P, Xu JH, Moreira D, Slatter M, Nademi Z, Worth A, Adams S, Jones A, Cale C, Allwood Z, Rao K, Chiesa R, Amrolia P, Gaspar H, Davies EG, Veys P, Gennery A, Qasim W (2014). Host natural killer immunity is a key indicator of permissiveness for donor cell engraftment in patients with severe combined immunodeficiency. J Allergy Clin Immunol.

[29] Dvorak CC, Hassan A, Slatter MA, Hönig M, Lankester AC, Buckley RH (2014). Comparison of outcomes of hematopoietic stem cell transplantation without chemotherapy conditioning by using matched sibling and unrelated donors for treatment of severe combined immunodeficiency. J Allergy Clin Immunol.

[30] Abd Hamid IJ, Slatter MA, McKendrick F, Pearce MS, Gennery AR. Long-Term Health Outcome and Quality of Life Post-HSCT for IL7Rα-, Artemis-, RAG1- and RAG2-Deficient Severe Combined Immunodeficiency: a Single Center Report. J *Clin Immunol*. 2018;38:727–32. 10.1007/s10875-018-0540-9 30105620

[31] Schuetz C, Neven B, Dvorak CC, Leroy S, Ege MJ, Pannicke U, Schwarz K, Schulz AS, Hoenig M, Sparber-Sauer M, Gatz SA, Denzer C, Blanche S, Moshous D, Picard C, Horn BN, de Villartay JP, Cavazzana M, Debatin KM, Friedrich W, Fischer A, Cowan MJ (2014). SCID patients with ARTEMIS vs RAG deficiencies following HCT: increased risk of late toxicity in ARTEMIS-deficient SCID. Blood.

[32] Haddad E, Logan BR, Griffith LM, Buckley RH, Parrott RE, Prockop SE (2018). SCID Genotype and 6-month Posttransplant CD4 Count Predict Survival and Immune Recovery. Blood.

[33] •• Punwani D, Kawahara M, Yu J, Sanford U, Roy S, Patel K, et al. Lentivirus Mediated Correction of Artemis-Deficient Severe Combined Immunodeficiency. *Hum Gene Ther*. 2017;28:112–24 **With reference 34, this describes lentiviral gene therapy as a potential treatment for Artemis-SCID.** 10.1089/hum.2016.064 PMC5278830 27611239

[34] •• Charrier S, Lagresle-Peyrou C, Poletti V, Rothe M, Cédrone G, Gjata B, et al. Biosafety Studies of a Clinically Applicable Lentiviral Vector for the Gene Therapy of Artemis-SCID. *Mol Ther Methods Clin Dev*. 2019;15:232–45 **With reference 33, this describes lentiviral gene therapy as a potential treatment for Artemis-SCID.** 10.1016/j.omtm.2019.08.014 PMC6838972 31720302

[35] Agarwal R, Dvorak CC, Proshaska S, Long-Boyle J, Kwon H-S, Brown JM (2019). Toxicity-free hematopoietic stem cell engraftment achieved with anti-CD117 monoclonal antibody conditioning. Biol Blood Marrow Transplant.

[36] van Os NJH, Haaxma CA, van der Flier M, Merkus PJFM, van Deuren M, de Groot IJM, et al. Ataxia-telangiectasia: recommendations for multidisciplinary treatment. Dev Med Child Neurol. 2017:59680–9. 10.1111/dmcn.13424 28318010

[37] Lefton-Greif MA, Winkelstein JA, Loughlin GM, Koerner CB, Zahurak M, Crawford TO (2000). Oropharyngeal dysphagia and aspiration in patients with ataxia-telangiectasia. J Pediatr.

[38] Sanal O, Ozbaş-Gerçeker F, Yel L, Ersoy F, Tezcan I, Berkel AI, Metin A, Gatti RA (2004). Defective anti-polysaccharide antibody response in patients with ataxia-telangiectasia. Turk J Pediatr.

[39] Reina-San-Martin B, Chen HT, Nussenzweig A, Nussenzweig MC (2004). ATM is required for efficient recombination between immunoglobulin switch regions. J Exp Med.

[40] Krauthammerr A, Lahad A, Goldberg L, Sarouk I, Weiss B, Somech R (2018). Elevated IgM levels as a marker for a unique phenotype in patients with Ataxia telangiectasia. BMC Pediatr.

[41] Amirifar P, Mozdarani H, Yazdani R, Kiaei F, Moeini Shad T, Shahkarami S, et al. Effect of Class Switch Recombination Defect on the Phenotype of Ataxia-Telangiectasia Patients. Immunol Invest 2020 **(in Press)**. 10.1080/08820139.2020.1723104 32116070

[42] Teraoka SN, Malone KE, Doody DR, Suter NM, Ostrander EA, Daling JR, Concannon P (2001). Increased frequency of ATM mutations in breast carcinoma patients with early onset disease and positive family history. Cancer.

[43] Rothblum-Oviatt C, Wright J, Lefton-Greif MA, McGrath-Morrow SA, Lederman HM, Crawford TO (2016). Ataxia telangiectasia: a review. Orphanet J Rare Dis.

[44] Suspitsin E, Sokolenko A, Bizin I, Tumakova A, Guseva M, Sokolova N, Vakhlyarskaya S, Kondratenko I, Imyanitov E (2020). ATM mutation spectrum in Russian children with ataxia-telangiectasia. Eur J Med Genet.

[45] Moin M, Aghamohammadi A, Kouhi A, Tavassoli S, Rezaei N, Ghaffari SR, Gharagozlou M, Movahedi M, Purpak Z, MirSaeid Ghazi B, Mahmoudi M, Farhoudi A (2007). Ataxia-telangiectasia in Iran: clinical and laboratory features of 104 patients. Pediatr Neurol.

[46] Suarez F, Mahlaoui N, Canioni D, Andriamanga C, Dubois d'Enghien C, Brousse N (2015). Incidence, presentation, and prognosis of malignancies in ataxia-telangiectasia: a report from the French national registry of primary immune deficiencies. J Clin Oncol.

[47] Crawford TO, Skolasky RL, Fernandez R, Rosquist KJ, Lederman HM (2006). Survival probability in ataxia telangiectasia. Arch Dis Child.

[48] van Os NJH, van Deuren M, Weemaes CMR, van Gaalen J, Hijdra H, Taylor AMR (2020). Classic ataxia-telangiectasia: the phenotype of long-term survivors. J Neurol.

[49] Zaki-Dizaji M, Akrami SM, Azizi G, Abolhassani H, Aghamohammadi A (2018). Inflammation, a significant player of Ataxia-Telangiectasia pathogenesis?. Inflamm Res.

[50] Hui CW, Song X, Ma F, Shen X, Herrup K (2018). Ibuprofen prevents progression of ataxia telangiectasia symptoms in ATM-deficient mice. J Neuroinflammation.

[51] Riabinska A, Lehrmann D, Jachimowicz RD, Knittel G, Fritz C, Schmitt A, Geyer A, Heneweer C, Wittersheim M, Frenzel LP, Torgovnick A, Wiederstein JL, Wunderlich CM, Ortmann M, Paillard A, Wößmann W, Borkhardt A, Burdach S, Hansmann ML, Rosenwald A, Perner S, Mall G, Klapper W, Merseburg A, Krüger M, Grüll H, Persigehl T, Wunderlich FT, Peifer M, Utermöhlen O, Büttner R, Beleggia F, Reinhardt HC (2020). ATM activity in T cells is critical for immune surveillance of lymphoma in vivo. Leukemia.

[52] Slack J, Albert MH, Balashov D, Belohradsky BH, Bertaina A, Bleesing J (2018). Inborn Errors Working Party of the European Society for Blood and Marrow Transplantation and the European Society for Immunodeficiencies; Stem Cell Transplant for Immunodeficiencies in Europe (SCETIDE); Center for International Blood and Marrow Transplant Research; Primary Immunodeficiency Treatment Consortium. Outcome of Hematopoietic Cell Transplantation for DNA Double-Strand Break Repair Disorders. J Allergy Clin Immunol.

[53] Duecker R, Baer PC, Buecker A, Huenecke S, Pfeffermann LM, Modlich U, Bakhtiar S, Bader P, Zielen S, Schubert R (2019). Hematopoietic Stem Cell Transplantation Restores Naïve T-Cell Populations in *Atm*-Deficient Mice and in Preemptively Treated Patients With Ataxia-Telangiectasia. Front Immunol.

[54] Bakhtiar S, Woelke S, Huenecke S, Kieslich M, Taylor AM, Schubert R, Zielen S, Bader P (2018). Pre-emptive Allogeneic Hematopoietic Stem Cell Transplantation in Ataxia Telangiectasia. Front Immunol.

[55] Ussowicz M, Wawrzyniak-Dzierżek E, Mielcarek-Siedziuk M, Salamonowicz M, Frączkiewicz J, Rybka B (2018). Allogeneic Stem Cell Transplantation after Fanconi Anemia Conditioning in Children with Ataxia-Telangiectasia Results in Stable T Cell Engraftment and Lack of Infections despite Mixed Chimerism. Biol Blood Marrow Transplant.

[56] Peffault de Latour R, Porcher R, Dalle JH, Aljurf M, Korthof ET, Svahn J, Willemze R, Barrenetxea C, Mialou V, Soulier J, Ayas M, Oneto R, Bacigalupo A, Marsh JC, Peters C, Socie G, Dufour C, FA Committee of the Severe Aplastic Anemia Working Party, Pediatric Working Party of the European Group for Blood and Marrow Transplantation (2013). Allogeneic hematopoietic stem cell transplantation in Fanconi anemia: the European Group for Blood and Marrow Transplantation experience. Blood.

[57] Mandola AB, Reid B, Sirror R, Brager R, Dent P, Chakroborty P (2019). Ataxia Telangiectasia Diagnosed on Newborn Screening-Case Cohort of 5 Years' Experience. Front Immunol.

[58] Mallott J, Kwan A, Church J, Gonzalez-Espinosa D, Lorey F, Tang LF (2013). Newborn screening for SCID identifies patients with ataxia telangiectasia. J Clin Immunol.

[59] Schoenaker MHD, Blom M, de Vries MC, Weemaes CMR, van der Burg M, Willemsen MAAP (2020). Early diagnosis of ataxia telangiectasia in the neonatal phase: a parents' perspective. Eur J Pediatr.

[60] Blom M, Schoenaker MHD, Hulst M, de Vries MC, Weemaes CMR, Willemsen MAAP, Henneman L, van der Burg M (2019). Dilemma of Reporting Incidental Findings in Newborn Screening Programs for SCID: Parents' Perspective on Ataxia Telangiectasia. Front Immunol.

[61] Weemaes CM, Hustinx TW, Scheres JM, van Munster PJ, Bakkeren JA, Taalman RD (1981). A new chromosomal instability disorder: the Nijmegen breakage syndrome. Acta Paediatr Scand.

[62] Wolska-Kusnierz B, Gregorek H, Chrzanowska K, Piatosa B, Pietrucha B, Heropolitanska-Pliszka E (2015). Nijmegen breakage syndrome: clinical and immunological features, long-term outcome and treatment options – a retrospective analysis. J Clin Immunol.

[63] Chrzanowska KH, Stumm M, Bekiesiska-Figatowska M, Varon R, Biaecka M, Gregorek H (2001). Atypical clinical picture of the Nijmegen breakage syndrome associated with developmental abnormalities of the brain. J Med Genet.

[64] Deripapa E, Balashov D, Rodina Y, Laberko A, Myakova N, Davydova NV, Gordukova MA, Abramov DS, Pay GV, Shelikhova L, Prodeus AP, Maschan MA, Maschan AA, Shcherbina A (2017). Prospective Study of a Cohort of Russian Nijmegen Breakage Syndrome Patients Demonstrating Predictive Value of Low Kappa-Deleting Recombination Excision Circle (KREC) Numbers and Beneficial Effect of Hematopoietic Stem Cell Transplantation (HSCT). Front Immunol.

[65] Marczak H, Heropolitańska-Pliszka E, Langfort R, Roik D, Grzela K (2018). Nijmegen breakage syndrome complicated with primary pulmonary granulomas. Pediatrics.

[66] Chrzanowska KH, Gregorek H, Dembowska-Bagińska B, Kalina MA, Digweed M (2012). Nijmegen breakage syndrome (NBS). J Orphanet Rare Dis.

[67] Patel JP, Puck JM, Srinivasan R, Brown C, Sunderam U, Kundu K (2015). Nijmegen breakage syndrome detected by newborn screening for T cell receptor excision circles (TRECs). J Clin Immunol.

[68] Dembowska-Baginska B, Perek D, Brozyna A, Wakulinska A, Olczak-Kowalczyk D, Gladkowska-Dura M, Grajkowska W, Chrzanowska KH (2009). Non-Hodgkin lymphoma (NHL) in children with Nijmegen breakage syndrome (NBS). Pediatr Blood Cancer.

[69] Albert MH, Gennery AR, Greil J, Cale CM, Kalwak K, Kondratenko I, Mlynarski W, Notheis G, Führer M, Schmid I, Belohradsky BH (2010). Successful SCT for Nijmegen breakage syndrome. Bone Marrow Transplant.

[70] Laberko A, Sultanova E, Gutovskaya E, Radygina S, Deripapa E, Kantulaeva A, et al. Treosulfan-based conditioning regimen in haematopoietic stem cell transplantation with TCRαβ/CD19 depletion in Nijmegen breakage syndrome. J Clin Immunol 2020 **(in press)**. 10.1007/s10875-020-00811-9 32602054

[71] Riballo E, Critchlow SE, Teo SH, Doherty AJ, Priestley A, Broughton B, Kysela B, Beamish H, Plowman N, Arlett CF, Lehmann AR, Jackson SP, Jeggo PA (1999). Identification of a defect in DNA ligase IV in a radiosensitive leukaemia patient. Curr Biol.

[72] van der Burg M, van Veelen LR, Verkaik NS, Wiegant WW, Hartwig NG, Barendregt BH, Brugmans L, Raams A, Jaspers NG, Zdzienicka MZ, van Dongen J, van Gent D (2006). A new type of radiosensitive T-B-NK+ severe combined immunodeficiency caused by a LIG4 mutation. J Clin Invest.

[73] Buck D, Moshous D, de Chasseval R, Ma Y, le Deist F, Cavazzana-Calvo M (2006). Severe combined immunodeficiency and microcephaly in siblings with hypomorphic mutations in DNA ligase IV. Eur J Immunol.

[74] Grunebaum E, Bates A, Roifman CM (2008). Omenn syndrome is associated with mutations in DNA ligase IV. J Allergy Clin Immunol.

[75] Toita N, Hatano N, Ono S, Yamada M, Kobayashi R, Kobayashi I, Kawamura N, Okano M, Satoh A, Nakagawa A, Ohshima K, Shindoh M, Takami T, Kobayashi K, Ariga T (2007). Epstein-Barr virus-associated B-cell lymphoma in a patient with DNA ligase IV (LIG4) syndrome. Am J Med Genet A.

[76] O'Driscoll M, Cerosaletti KM, Girard PM, Dai Y, Stumm M, Kysela B, Hirsch B, Gennery A, Palmer SE, Seidel J, Gatti RA, Varon R, Oettinger MA, Neitzel H, Jeggo PA, Concannon P (2001). DNA ligase IV mutations identified in patients exhibiting developmental delay and immunodeficiency. Mol Cell.

[77] Staines Boone AT, Chinn IK, Alaez-Versón C, Yamazaki-Nakashimada MA, Carrillo-Sánchez K, García-Cruz MLH (2019). Failing to Make Ends Meet: The Broad Clinical Spectrum of DNA Ligase IV Deficiency. Case Series and Review of the Literature. Front Pediatr.

[78] Felgentreff K, Baxi SN, Lee YN, Dobbs K, Henderson LA, Csomos K (2016). Ligase-4 Deficiency Causes Distinctive Immune Abnormalities in Asymptomatic Individuals. J Clin Immunol.

[79] Murray JE, Bicknell LS, Yigit G, Duker AL, van Kogelenberg M, Haghayegh S, Wieczorek D, Kayserili H, Albert MH, Wise CA, Brandon J, Kleefstra T, Warris A, van der Flier M, Bamforth JS, Doonanco K, Adès L, Ma A, Field M, Johnson D, Shackley F, Firth H, Woods CG, Nürnberg P, Gatti RA, Hurles M, Bober MB, Wollnik B, Jackson AP (2014). Extreme growth failure is a common presentation of ligase IV deficiency. Hum Mutat.

[80] Bacon CM, Wilkinson SJ, Spickett GP, Barge D, Lucraft HH, Jackson G, et al. Epstein-Barr virus-independent diffuse large B-cell lymphoma in DNA ligase 4 deficiency. *J Allergy Clin Immunol* 2013;131:1237–9, 1239.e1. 10.1016/j.jaci.2012.10.027 23228243

[81] Buck D, Malivert L, de Chasseval R, Barraud A, Fondanèche M-C, Sanal O (2006). Cernunnos, a novel nonhomologous end-joining factor, is mutated in human immunodeficiency with microcephaly. Cell.

[82] Ahnesorg P, Smith P, Jackson SP (2006). XLF interacts with the XRCC4-DNA ligase IV complex to promote DNA nonhomologous end-joining. Cell.

[83] Dai Y, Kysela B, Hanakahi LA, Manolis K, Riballo E, Stumm M, Harville TO, West SC, Oettinger MA, Jeggo PA (2003). Nonhomologous end joining and V(D)J recombination require an additional factor. Proc Natl Acad Sci U S A.

[84] Patiroglu T, Akar HH, van der Burg M, Kontas O (2015). A Case of XLF Deficiency Presented With Diffuse Large B Cell Lymphoma in the Brain. Clin Immunol.

[85] van der Burg M, Ijspeert H, Verkaik NS, Turul T, Wiegant WW, Morotomi-Yano K, Mari PO, Tezcan I, Chen DJ, Zdzienicka MZ, van Dongen J, van Gent D (2009). A DNA-PKcs mutation in a radiosensitive T-B- SCID patient inhibits Artemis activation and nonhomologous end-joining. J Clin Invest.

[86] Woodbine L, Neal JA, Sasi NK, Shimada M, Deem K, Coleman H, Dobyns WB, Ogi T, Meek K, Davies EG, Jeggo PA (2013). PRKDC mutations in a SCID patient with profound neurological abnormalities. J Clin Invest.

[87] Mathieu AL, Verronese E, Rice GI, Fouyssac F, Bertrand Y, Picard C (2015). PRKDC mutations associated with immunodeficiency, granuloma, and autoimmune regulator-dependent autoimmunity. J Allergy Clin Immunol.

[88] Esenboga S, Akal C, Karaatmaca B, Erman B, Dogan S, Orhan D (2018). Two siblings with PRKDC defect who presented with cutaneous granulomas and review of the literature. Clin Immunol.

[89] Berland A, Rosain J, Kaltenbach S, Allain V, Mahlaoui N, Melki I (2019). PROMIDISα: A T-cell receptor α signature associated with immunodeficiencies caused by V(D)J recombination defects. J Allergy Clin Immunol.

[90] Huang X, Darzynkiewicz Z (2006). Cytometric assessment of histone H2AX phosphorylation: a reporter of DNA damage. Methods Mol Biol.

[91] Buchbinder D, Smith MJ, Kawahara M, Cowan MJ, Buzby JS, Abraham RS (2018). Application of a Radiosensitivity flow assay in a patient with DNA ligase 4 deficiency. Blood Adv.

[92] Slatter MA, Gennery AR (2010). Primary immunodeficiencies associated with DNA-repair disorders. Expert Rev Mol Med.

[93] Fiévet A, Bellanger D, Valence S, Mobuchon L, Afenjar A, Giuliano F (2019). Three New Cases of Ataxia-Telangiectasia-Like Disorder: No Impairment of the ATM Pathway, but S-phase Checkpoint Defect. Hum Mutat.

[94] Lähdesmäki A, Taylor AMR, Chrzanowska KH, Pan-Hammarström Q (2004). Delineation of the role of the Mre11 complex in class switch recombination. J Biol Chem.

[95] Rosin N, Elcioglu NH, Beleggia F, Isguven P, Altmuller J, Thiele H (2015). Mutations in XRCC4 cause primary microcephaly, short stature and increased genomic instability. Hum Mol Genet.

[96] Murray JE, van der Burg M, Uspeert H, Carroll P, Wu Q, Ochi T (2015). Mutations in the NHEJ component XRCC4 cause primordial dwarfism. Am J Hum Genet.

[97] de Bruin C, Mericq V, Andrew SF, van Duyvenvoorde HA, Verkaik NS, Losekoot M, Porollo A, Garcia H, Kuang Y, Hanson D, Clayton P, van Gent DC, Wit JM, Hwa V, Dauber A (2015). An XRCC4 splice mutation is associated with severe short stature, gonadal failure, and early-onset metabolic syndrome. J Clin Endocrinol Metab.

[98] Bee L, Nasca A, Zanolini A, Cendron F, d’Adamo P, Costa R (2015). A nonsense mutation of human XRCC4 is associated with adult-onset progressive encephalocardiomyopathy. EMBO Mol Med.

[99] Guo C, Nakazawa Y, Woodbine L, Björkman A, Shimada M, Fawcett H, Jia N, Ohyama K, Li TS, Nagayama Y, Mitsutake N, Pan-Hammarström Q, Gennery AR, Lehmann AR, Jeggo PA, Ogi T (2015). XRCC4 deficiency in human subjects causes a marked neurological phenotype but no overt immunodeficiency. J Allergy Clin Immunol.

[100] Bomken S, van der Werff Ten Bosch J, Attarbaschi A, Bacon CM, Borkhardt A, Boztug K (2018). Current Understanding and Future Research Priorities in Malignancy Associated With Inborn Errors of Immunity and DNA Repair Disorders: The Perspective of an Interdisciplinary Working Group. Front Immunol.

